# Cross-study analysis of gene expression data for intermediate neuroblastoma identifies two biological subtypes

**DOI:** 10.1186/1471-2407-7-89

**Published:** 2007-05-25

**Authors:** Patrick Warnat, André Oberthuer, Matthias Fischer, Frank Westermann, Roland Eils, Benedikt Brors

**Affiliations:** 1 Department of Theoretical Bioinformatics (B080), German Cancer Research Center (DKFZ), Im Neuenheimer Feld 280, D-69120 Heidelberg, Germany; 2 Children's Hospital, Department of Pediatric Oncology and Hematology and Center for Molecular Medicine Cologne (CMMC), University of Cologne, Kerpener Strasse 62, D-50924 Cologne, Germany; 3 Department of Tumor Genetics (B030), German Cancer Research Center (DKFZ), Im Neuenheimer Feld 280, D-69120 Heidelberg, Germany

## Abstract

**Background:**

Neuroblastoma patients show heterogeneous clinical courses ranging from life-threatening progression to spontaneous regression. Recently, gene expression profiles of neuroblastoma tumours were associated with clinically different phenotypes. However, such data is still rare for important patient subgroups, such as patients with *MYCN *non-amplified advanced stage disease. Prediction of the individual course of disease and optimal therapy selection in this cohort is challenging. Additional research effort is needed to describe the patterns of gene expression in this cohort and to identify reliable prognostic markers for this subset of patients.

**Methods:**

We combined gene expression data from two studies in a meta-analysis in order to investigate differences in gene expression of advanced stage (3 or 4) tumours without *MYCN *amplification that show contrasting outcomes (alive or dead) at five years after initial diagnosis. In addition, a predictive model for outcome was generated. Gene expression profiles from 66 patients were included from two studies using different microarray platforms.

**Results:**

In the combined data set, 72 genes were identified as differentially expressed by meta-analysis at a false discovery rate (FDR) of 8.33%. Meta-analysis detected 34 differentially expressed genes that were not found as significant in either single study. Outcome prediction based on data of both studies resulted in a predictive accuracy of 77%. Moreover, the genes that were differentially expressed in subgroups of advanced stage patients without *MYCN *amplification accurately separated *MYCN *amplified tumours from low stage tumours without *MYCN *amplification.

**Conclusion:**

Our findings support the hypothesis that neuroblastoma consists of two biologically distinct subgroups that differ by characteristic gene expression patterns, which are associated with divergent clinical outcome.

## Background

Neuroblastoma is a malignant tumour of the sympathetic nervous system. Next to brain tumours, it is the most common solid tumour in children, with 7.5 cases per 100,000 infants [1]. The hallmark of this tumour is its heterogeneous clinical behaviour, ranging from life-threatening tumour progression to spontaneous regression or differentiation to benign ganglioneuroma. To discriminate these contrasting patterns of clinical behaviour, several molecular and cytogenetic features, such as amplification of the *MYCN *oncogene or deletion of chromosomal material from 1p or 11q are currently used in clinical trials [1,2]. However, the course of intermediate risk patients with *MYCN *non-amplified advanced stage disease is still hard to predict by these markers, and optimal therapy selection in this cohort remains challenging. Thus, current neuroblastoma trials may stratify these advanced stage *MYCN *non-amplified patients to either the low-, the intermediate- or the high-risk group leading to highly differing treatment approaches [3]. Therefore, additional research effort is needed to identify reliable prognostic markers for these subsets of patients [4].

To this end, recent studies applied gene expression profiling to investigate divergent clinical neuroblastoma phenotypes [5-8]. These studies have shown that it is possible to discriminate subtypes of neuroblastomas of diverse molecular and clinical phenotype by their gene expression profiles. However, gene expression data for the important subgroup of *MYCN *non-amplified neuroblastoma patients of advanced stage disease (International Neuroblastoma Staging System, INSS Stage 3 or 4,) is still rare, leading to a high risk of false positive and false negative findings [9].

In this study, we combined gene-expression data from two different studies generated by different platforms, since a combined analysis yields more information than each individual study [10,11]. Although microarray data generated by different platforms are not directly comparable, they can be combined in an integrative analysis, if appropriate methods are carefully chosen [12]. We focused on *MYCN *non-amplified tumours with advanced INSS stage 3 or 4. We searched for differences in gene expression between patients with contrasting outcomes (alive or dead) at five years after initial diagnosis. Differentially expressed genes were identified and described based on data of the two largest neuroblastoma gene expression studies to date [7,8]. In addition, a predictive model (classifier) for patient outcome was generated and assessed.

## Methods

### Gene Expression Data

Microarray data by Ohira et al. [7] were downloaded from the NCBI GEO [13] database (accession number GSE2283), and microarray data by Oberthuer et al. [8] were downloaded from the EBI ArrayExpress [14] database (accession number E-TABM-38). For both data sets normalized expression data were used as published.

The probes of the different microarray platforms were mapped by a perfect match of their sequences. If an oligonucleotide probe of the platform used by Oberthuer et al. had a sequence that could be matched to a part of the GenBank mRNA sequence corresponding to a cDNA clone probe of the platform used by Ohira et al., both probes were considered as representing the same transcript. For genes represented by several probes on a platform the median expression value of all corresponding probes was used.

Gene expression profiles of patients were selected from both studies according to the following criteria: *MYCN *non-amplified tumors with advanced INSS stage (3 or 4) with either a minimum follow-up time of five years after diagnosis or with fatal outcome of disease. All patients were grouped according to their outcome status (alive vs. dead) five years after initial diagnosis. In addition, we randomly selected 40 other patients from both studies (*MYCN *amplified stage 3 or 4 tumours, ten of each study; *MYCN *non amplified tumours with stages 1, 2 or 4S, ten of each study). According to their clinical characteristics, these patients have either a highly aggressive neuroblastoma tumour or a tumour with very good prognosis. We used the microarray data of these tumors to study the expression of the genes found to be differentially expressed in our meta-analysis of intermediate risk neuroblastomas in patients with either distinct favourable or unfavourable course of disease.

### Identification of significant genes

To detect significant differential expression of a gene between the two outcome patient groups across studies, we applied a meta-analysis approach as described by Choi et al. [10]. The Bioconductor [15] software package GeneMeta was used to perform these calculations.

Moreover, to explore the biological aspects of the significant genes, we analysed the associated Gene Ontology (GO) [16] terms using the GOstat software [17], which generates statistics of which annotations are overrepresented in a given list of genes.

### Classification analysis

A predictive model for the patient outcome status (alive vs. dead) five years after diagnosis was generated using the method of nearest shrunken centroids classification [18].

Using only the data of genes represented on both microarray platforms (n = 1,271), we applied a methodology evaluated by Warnat et al. [11], namely the median rank scores (MRS), to derive numerically comparable quantities from the expression values of both platforms used in the different studies. In total, 36 patients were selected from both studies as a training set to generate a predictive model of patient outcome (Additional File 1). The remaining 30 patients were used as an independent test set. In addition, samples of the independent test set were classified according to the clinical markers age at diagnosis and INSS stage.

Supplementary information is available at the website of BMC Cancer.

## Results

### Gene Expression Data

We focused on data of the two biggest current neuroblastoma gene-expression studies from Ohira et al. [7] and Oberthuer et al. [8]. These studies analysed a total of 136 (Ohira) and 251 patients (Oberthuer). Further neuroblastoma gene expression studies by Wei et al. [5] and Schramm et al. [6] comprised 18 and 4 patients that met the criteria of our cohort (patients with advanced stage disease without *MYCN*-amplification). However, based on mappings to the Unigene database, the number of UniGene clusters represented on each of the four microarray platforms used in the studies of Wei et al., Schramm et al., Ohira et al. and Oberthuer et al. only sums up to 362 UniGene clusters. In addition, the data of Wei et al. is not available in a public microarray data repository. Thus, we decided to use only data from the studies of Ohira et al. and Oberthuer et al.

In the study of Ohira et al. [7] a cDNA microarray platform with 5,430 probes representing 4,204 different GenBank entries was used. In the study of Oberthuer et al. [8] an oligonucleotide array with 10,163 probes representing 8,155 UniGene clusters was used. By means of sequence comparison, we found 1,271 genes that are represented on both platforms. Applying a minimum follow-up time of five years after initial diagnosis, gene expression profiles of *MYCN *non-amplified tumours with advanced INSS stage (3 or 4) from 66 patients were obtained in total from both studies (Table 1).

**Table 1:** Patient characteristics.

***Study***	***Oberthuer et al.***	***Ohira et al.***
*Outcome*		
F	27	14
UF	16	9
		
*Age*		
<1a	31	9
>1a	12	14
		
*Stage*		
3	13	13
4	30	10
		
*1p*		
Het	31	n.d.
Im	5	n.d.
Del	6	n.d.
ND	1	n.d.

For the study of Ohira et al. no information about the 1p status was available(n.d.: not determined). F: favourable; UF: unfavourable; Het: heterozygous; Im: imbalance; Del: deletion; ND: not defined.

Although risk stratification and treatment strategies of neuroblastoma are different for a small fraction of patients [ref. [3] and Additional File 1] between Japan and Germany, the resulting 5-years overall survival is comparable among the German and Japanese neuroblastoma risk groups, suggesting that the selected patient cohorts from both studies [7,8] can be compared in a combined analysis as performed here.

### Identification of significant genes

To detect significant differential expression of a gene between the two outcome patient groups across studies, we applied a meta-analysis approach as described by Choi et al. [10]. Based on a Q statistic it was decided to use a random effects model in the meta-analysis rather than a fixed effects model (Additional File 1). Using a random effects model and a threshold of 2.57 (corresponding to p = 0.01 of a N(0,1) distribution) for the average effect size calculated on both data sets, we identified 72 genes which are significantly differentially expressed between the two outcome groups at a false discovery rate of 8.33%. A number of 34 of these were found exclusively in the meta-analysis of both sets, and not in any single study data-based analysis applying the same threshold (Table 2). This finding points to the fact that meta-analyses, due to an enhanced statistical power, may disclose differentially expressed genes that are missed by analyses of single study gene expression data sets.

**Table 2:** List of differentially expressed genes.

*Symbol*	*Name*	*z score*	*only found in M.A.*
NDUFV1	NADH dehydrogenase (ubiquinone) flavoprotein 1, 51 kDa	-3.98	
TP53	Tumor protein p53 (Li-Fraumeni syndrome)	-3.54	
AHCY	S-adenosylhomocysteine hydrolase	-3.44	
NPM1	Nucleophosmin (nucleolar phosphoprotein B23, numatrin)	-3.38	*
CHD1L	Chromodomain helicase DNA binding protein 1-like	-3.26	
CCNB1	Cyclin B1	-3.22	
HSPA5	Heat shock 70 kDa protein 5 (glucose-regulated protein, 78 kDa)	-3.20	
SF4	Splicing factor 4	-3.19	
CCNA2	Cyclin A2	-3.15	
RPS13	Ribosomal protein S13	-3.14	*
E2F1	E2F transcription factor 1	-3.12	
FLJ11806	Nuclear protein UKp68	-3.12	
RUVBL1	RuvB-like 1 (E. coli)	-3.10	
CCT5	Chaperonin containing TCP1, subunit 5 (epsilon)	-3.05	*
CCT3	Chaperonin containing TCP1, subunit 3 (gamma)	-3.02	
DDX49	DEAD (Asp-Glu-Ala-Asp) box polypeptide 49	-2.89	*
EIF2S1	Eukaryotic translation initiation factor 2, subunit 1 alpha, 35kDa	-2.88	
KIFC1	Kinesin family member C1	-2.88	*
FLJ13910	Hypothetical protein FLJ13910	-2.85	
APEX1	APEX nuclease (multifunctional DNA repair enzyme) 1	-2.83	
COPB	Coatomer protein complex, subunit beta	-2.83	
CDC25B	Cell division cycle 25B	-2.81	*
TMED2	transmembrane emp24 domain trafficking protein 2	-2.73	
AHCYL1	S-adenosylhomocysteine hydrolase-like 1	-2.71	*
RAD23A	RAD23 homolog A (S. cerevisiae)	-2.70	*
HMGB2	High-mobility group box 2	-2.67	
TRIM28	Tripartite motif-containing 28	-2.64	*
ENO1	Enolase 1, (alpha)	-2.62	*
MRPL3	Mitochondrial ribosomal protein L3	-2.61	*
MARK2	MAP/microtubule affinity-regulating kinase 2	-2.61	*
DDX1	DEAD (Asp-Glu-Ala-Asp) box polypeptide 1	-2.59	*
KIF22	Kinesin family member 22	-2.57	
MLL	myeloid/lymphoid or mixed-lineage leukemia (trithorax homolog, Drosophila)	2.57	*
YWHAE	Tyrosine 3-monooxygenase/tryptophan 5-monooxygenase activation protein	2.58	
DUSP16	Dual specificity phosphatase 16	2.62	*
EZH1	Enhancer of zeste homolog 1 (Drosophila)	2.63	*
NCAM1	Neural cell adhesion molecule 1	2.63	*
MAP2K4	Mitogen-activated protein kinase kinase 4	2.64	
ELAVL4	ELAV (embryonic lethal, abnormal vision, Drosophila)-like 4 (Hu antigen D)	2.65	*
RBMS3	RNA binding motif, single stranded interacting protein	2.65	*
PXK	PX domain containing serine/threonine kinase	2.66	*
PMSCL2	Exosome component 10	2.67	*
NTRK1	Neurotrophic tyrosine kinase, receptor, type 1	2.67	*
DOCK4	Dedicator of cytokinesis 4	2.70	*
AB051522	DIX domain containing 1	2.70	*
RAB2	RAB2, member RAS oncogene family	2.70	*
FOXP1	Forkhead box P1	2.74	*
GPS2	G protein pathway suppressor 2	2.79	*
MLL5	Myeloid/lymphoid or mixed-lineage leukemia 5 (trithorax homolog, Drosophila)	2.82	
DLG4	Discs, large homolog 4 (Drosophila)	2.84	*
VAMP2	Vesicle-associated membrane protein 2 (synaptobrevin 2)	2.85	
NXPH1	Neurexophilin 1	2.89	*
ATP6V1A	ATPase, H+ transporting, lysosomal 70 kDa, V1 subunit A	2.91	
ZNF218	zinc finger protein 218	2.93	
FLJ13110	Receptor accessory protein 1	2.94	
CDK5R1	Cyclin-dependent kinase 5, regulatory subunit 1 (p35)	2.96	*
NCOA7	nuclear receptor coactivator 7	2.96	*
FLJ11730	Chromosome 1 open reading frame 149	3.04	*
MARCKS	Myristoylated alanine-rich protein kinase C substrate	3.06	*
CLSTN3	Calsyntenin 3	3.08	
CAMTA1	Calmodulin binding transcription activator 1	3.10	
SCN3B	sodium channel, voltage-gated, type III, beta	3.11	*
PTN	Pleiotrophin (heparin binding growth factor 8, neurite growth-promoting factor 1)	3.14	
EPS15	Epidermal growth factor receptor pathway substrate 15	3.26	
BRUNOL4	Bruno-like 4, RNA binding protein (Drosophila)	3.26	
DCAMKL1	Doublecortin and CaM kinase-like 1	3.30	
ALS2CL	ALS2 C-terminal like	3.38	
LOC284244	Hypothetical protein LOC284244	3.41	
RAPGEF6	Rap guanine nucleotide exchange factor (GEF) 6	3.42	
PKIB	Protein kinase (cAMP-dependent, catalytic) inhibitor beta	3.50	
LOC116236	Hypothetical protein LOC116236	3.89	
FYN	FYN oncogene related to SRC, FGR, YES	3.96	

Column '*only found in M.A*.': Those genes are marked that were exclusively identified by meta-analysis. A negative z-score indicates down-regulation of a gene in tumors with a favourable prognosis relative to the expression in tumors with unfavourable prognosis.

**Figure 1:**
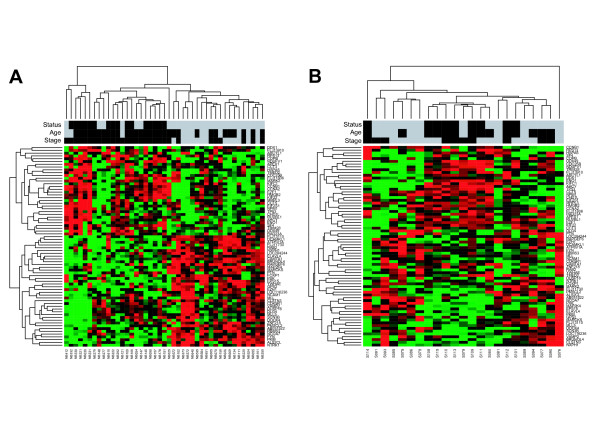
**Hierarchical clustering of the expression data for the significant genes in advanced stage *MYCN *non-amplified tumors**. (A) Oligonucleotide data. (B) cDNA data. The colored bars at the top of the figure denote the values of the following clinical variables: Status: black – unfavourable outcome, grey – favourable outcome; Age (at diagnosis): black – older than one year, grey – younger than one year; Stage: black – INSS stage 4, grey – INSS stage 3.

To visualise the expression of the significant genes, colour maps were generated showing a hierarchical clustering of the gene expression values for each study separately (Fig. 1A,B). The samples of the study of Oberthuer et al. (Figure 1A) are grouped into threee main groups, as indicated by the sample dendrogram generated by hierarchical clustering. These three groups consist of the following samples (from left to right): NB412-NB331, NB279-NB025, NB572-NB250. The two leftmost groups mainly contain patients with unfavourable outcome, while only seven of 23 patients in these two groups show a favourable outcome. Of these seven patients, three already had a clinical event (see following paragraph about clinical courses of these patients). The third group (NB572-NB250) contains 20 patients, all having a favourable outcome. In the clustering results of Ohira et al., two samples can be identified that show an expression profile clearly distinct from the other samples: S114 and S076. From the sample dendrogram and the heatmap, three groups can be derived for all remaining samples (from left to right): S091-S078, S108-S080, S81-S90. The second group (S108-S080) mainly contains patients with unfavourable outcome (six out of eight), whereas the two other groups contain mainly patients with favourable outcome (11 of 13). Thus, most of the samples with similar clinical outcome are grouped together, with a better grouping resulting from the data of Oberthuer et al. Furthermore, the set of differentially expressed genes can also be divided into two groups according to their expression, one group shows low expression in tumours with favourable outcome and high expression in patients with an unfavourable outcome while the other group shows inverted expression behaviour. The gene dendrograms of the heatmaps in figure 1A and 1B both show a top level split that yields two roughly equal-sized groups of 32 and 40 genes (Fig. 1A) or 30 and 42 genes (Fig. 1B), respectively. The set of genes in these two groups are very similar in the two heatmaps for the different studies, as only two genes (*TMED2 *and *MARK2*) are grouped differently in the respective other study. Thus, the set of 72 can be divided into two groups in each of which genes show a highly consistant gene expression behaviour.

**Figure 2:**
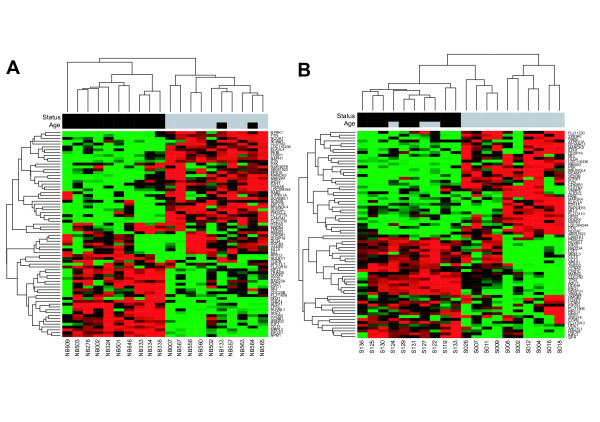
**Hierarchical clustering of the expression data for the significant genes in advanced stage *MYCN *amplified tumors versus low stage non-amplified tumors**. (A) Oligonucleotide data. (B) cDNA data. The colored bars at the top of the figure denote the values of the following clinical variables: Status: black – unfavourable outcome, grey – favourable outcome; Age (at diagnosis): black – older than one year, grey – younger than one year.

By generating further colour maps for the expression of the selected genes in *MYCN *amplified stage 3 or 4 tumours and *MYCN *non amplified tumours with stages 1, 2, 4s of both studies (Fig. 2A,B), it was observed that the expression pattern of the genes selected by meta-analysis of intermediate risk neuroblastoma explicitly differs in the most aggressive tumours compared to tumours with very good prognosis. The full set of tumours from both studies is shown in Additional Files 2 and 3. The tumours with clearly defined prognosis show similar gene expression patterns for the set of 72 differentially expressed genes as the investigated tumour subgroup. These results suggest that all *MYCN *non-amplified tumours with advanced stage (3 or 4) can be distinguished into just two biological subtypes with contrasting clinical outcome according to the expression profile of the 72 differentially expressed genes. Moreover, expression of these genes also seems to be a marker for tumour aggressiveness in other tumour subgroups.

### Clinical courses observed in non-amplified advanced stage patients with differing gene-expression patterns

In the data set of Oberthuer et al., seven patients that were alive five years after diagnosis were clustered together with those patients that had succumbed to disease (Fig. 1A). Three of them had experienced an event, two of them even had been treated with intensive salvage therapy, but all are currently in complete or incomplete remission. Opposed to that, three patients were documented to have experienced an event within the subset of patients that were clustered together as being alive five years after diagnosis. In all these patients, local tumour progression was diagnosed but either surgical intervention revealed maturation of the tissue or medium-dose chemotherapy resulted in event-free survival since then. Thus, a different quality of events was observed in those patients whose gene-expression profile resembles that of patients who succumbed to disease, as opposed to those events that were observed in patients whose gene-expression pattern matched long-term event-free survivors. For the data set of Ohira et al. (Fig. 1B) no detailed information about clinical history of investigated patients was available.

### Biological roles of the differentially expressed genes

Using the set of 72 significant genes for a hierarchical clustering of *MYCN *amplified stage 3 or 4 tumours and *MYCN *non-amplified tumours with stages 1, 2, 4s, both prognosis groups are clearly separated (Fig. 2). Therefore, the expression of these 72 genes seems to be of general importance for distinction of prognosis groups in neuroblastoma tumours.

In the group of genes that shows high expression in tumours with favourable outcome, genes related to cell differentiation (*FYN*, *NTRK1*) and neuronal development (*DCAMKL1*) can be found. Among the significant genes that show a high expression in tumours with unfavourable outcome, cell cycle associated genes can be found (*E2F1*, *CCNA2*, *CCNB1*, *KIFC1*).

To further characterize which biological functions are defined by these genes, we explored the GO terms associated with the significant genes in order to identify the biological roles represented by these genes. We ranked the GO terms associated with the selected genes in our meta-analysis by using the p-values generated in a GO based gene set enrichment analysis as implemented in the GOstat tool. The most highly ranked annotation terms are listed in Table 3. Among these, GO terms can be found for which the associated genes all show higher expression in tumours with unfavourable outcome (e.g. cell cycle and DNA maintenance associated terms), as well as terms for which the associated genes show higher expression in tumours with favourable outcome (e.g. negative regulation of MAPK activity). The genes associated with the given GO terms are listed in Additional File 4.

**Table 3:** Enriched GO terms in the category "Biological Process" among the significantly differentially expressed genes.

*GO term*	*Expression*	*Count {55}*	*Total {857}*	*p-value*	*Corrected p*
base-excision repair	-	2	2	0.004	0.307
regulation of kinase activity	+	5	19	0.005	0.307
regulation of protein kinase activity	+	5	19	0.005	0.307
regulation of transferase activity	+	5	20	0.007	0.307
negative regulation of transferase activity	+	3	7	0.007	0.307
negative regulation of protein kinase activity	+	3	7	0.007	0.307
negative regulation of enzyme activity	+	3	8	0.011	0.361
regulation of enzyme activity	+	5	25	0.018	0.361
response to DNA damage stimulus	-	4	17	0.019	0.361
response to endogenous stimulus	-	4	17	0.019	0.361
MAPKKK cascade	+	3	10	0.022	0.361
one-carbon compound metabolism	-	2	4	0.022	0.361
negative regulation of MAPK activity	+	2	4	0.022	0.361
inactivation of MAPK activity	+	2	4	0.022	0.361
M phase of mitotic cell cycle	-	4	18	0.024	0.361
phosphorus metabolism	+	9	68	0.033	0.361
phosphate metabolism	+	9	68	0.033	0.361
M phase	-	4	20	0.034	0.361
regulation of cyclin dependent protein kinase activity	-	2	5	0.036	0.361
glycolysis	-	2	5	0.036	0.361
Wnt receptor signaling pathway	+	2	5	0.036	0.361
oxidative phosphorylation	*	2	5	0.036	0.361

Expression: expression of associated genes in tumors with a favourable prognosis relative to the expression in tumors with unfavourable prognosis; +: up-regulation; -: down-regulation; *: genes show oppositional expression.

Count: number of significant genes associated with given GO term. Total: number of genes in analysis associated with given GO term. The numbers in curly brackets denote the number of genes that could be mapped to GO. Corrected p-values were calculated according to Benjamini and Hochberg as implemented in GOstat.

### Classification analysis

According to the German neuroblastoma risk stratification protocol, patients with non-*MYCN *amplified advanced stage 3 or 4 disease may be grouped into either the low-, the intermediate- or the high-risk group, depending on their age at diagnosis, or an alteration of the short arm of chromosome 1 (del1p). Therefore, the investigated subgroup of neuroblastoma patients would greatly profit from an improved risk estimation. Independently to the analysis described above, we generated a predictive model for patient outcome using the method of nearest shrunken centroids classification [18] based on data of both studies. For this, 36 patients were selected from both studies as a training set, the remainig patients were used as a independent test set. The resulting predictive model, achieved a predictive accuracy of 77% for neuroblastoma in stage 3 or 4 without *MYCN *amplification in the independent test set (Table 4), using the expression values of 256 genes. Inferior predictive accuracies were achieved when the samples were classified by the clinical parameters age and stage, although no statistical significance of the difference in predictive accuracy between the best clinical parameter (a combination of age and stage) and the microarray based classification was observed, as the 95% confidence intervals were overlapping. Application of the microarray based classifier also resulted in good performance in terms of both sensitivity and specificity, indicating the feasibility of generating and applying a predictive model of outcome based on data of different gene expression profiling studies. The overlap of the list of 256 genes used for classification with the list of 72 genes identified by meta analysis and the list of 144 genes used for classification in [8] is shown in Additional File 5.

**Table 4:** Performance of different markers for outcome prediction on the independent test set.

	*Accuracy*	*Sensitivity*	*Specificity*
Microarray classifier	0.77, [0.66;0.85], (23/30)	0.86, [0.62;0.96], (6/7)	0.74, [0.61;0.84], (17/23)
Age	0.53, [0.42;0.65], (16/30)	1.0, [0.81;1.0], (7/7)	0.39, [0.27;0.52], (9/23)
Stage	0.60, [0.48;0.71], (18/30)	1.0, [0.81;1.0], (7/7)	0.48, [0.35;0.61], (11/23)
Age + Stage	0.70, [0.58;0.79], (21/30)	1.0, [0.81;1.0], (7/7)	0.61, [0.48;0.73], (14/23)

The figures in brackets denote 95% confidence intervals and absolute number of cases. For prediction of outcome by age at diagnosis, patients older than one year were predicted with unfavourable outcome. For prediction by stage, patients with stage 3 were predicted with favourable outcome and patients with stage 4 were predicted with unfavourable outcome. For the combination of the markers age and stage, only stage 4 patients older than one year at dignosis were predicted with unfavourable outcome. Sensitivity/Specificity is rate of correctly predicted patients with unfavourable/favourable outcome.

## Discussion

In this study, we combined gene expression data from two different studies to investigate the differences in gene expression for advanced stage *MYCN *non-amplified tumours with contrasting outcome at five years after initial diagnosis.

Our results suggest that this subgroup of tumours can be distinguished into two biological subtypes showing distinct gene expression profiles that are associated with contrasting clinical outcomes. The expression of the genes that are differentially expressed between these two subtypes may represent a general indicator of neuroblastoma aggressiveness, since corresponding expression behaviour can be observed in low stage *MYCN *non-amplified tumours as well as advanced stage *MYCN *amplified tumours.

Instead of simply comparing lists of differentially expressed genes obtained on single study data or combining p-values calculated for each single study [19], we applied a method of meta-analysis on the gene expression data that is based on a well established statistical framework and comprises modelling of study-to-study differences [10]. Unfortunately, the combination of gene expression data from different studies has the disadvantage that only genes common to all microarray platforms can be used. As the reliability of the probe mapping is crucial for a cross-platform analysis, we applied a stringent sequence based mapping of the probes of different microarray platforms in order to avoid inappropriate mapping of the probes. The combination of data from different studies for our analysis resulted in a large number of included expression profiles for the investigated subset of patients although a stringent selection criterion of a follow up of 5 years was applied. This clearly led to a higher statistical power of our analysis in comparison to single study based results, since of the 72 significantly differentially expressed genes, 34 genes were found exclusively in the meta-analysis of both sets.

Among the significantly differentially expressed genes are some that are known in the context of neuroblastoma research. Our results confirm observations made for these genes as described earlier in literature. High expression of *NTRK1 *is present in neuroblastomas with favourable biological features and highly correlated with patient survival [20]. High expression of *FYN *and high FYN kinase activity are restricted to low-stage tumours [21]. *PTN *is highly expressed in favourable neuroblastomas, whereas it is expressed at a significantly lower level in advanced tumours [22]. Low *CAMTA1 *expression is associated with poor outcome [23]. *NCAM *expression seems to enhance the malignancy of neuroblastoma cells and their tendency to metastasise [24]. High *HuD *(*ELAVL4*) mRNA levels may predict a clinically favourable outcome [25]. The fact that some of these genes were exclusively detected by the meta-analysis (Table 2) underlines the benefit of cross-study analyses for investigation of tumour subgroups. Interestingly, the 72 genes found to be significant for the investigated subgroup of neuroblastoma tumours also show a distinct differential expression in other prognostic subgroups and may thus be used as a general prognostic marker for neuroblastoma patients. Moreover, this result suggests that, although for neuroblastoma tumours several different clinical stages and risk groups are defined, on the level of gene expression they seem to comprise only two distinct biological entities associated with adverse patient outcome.

The GO based gene set enrichment analysis of the GO terms associated with the selected genes in our meta-analysis did not show that any GO term is overrepresented with high statistical significance. However, the p-values calculated in the gene set enrichment analysis provide a useful ranking of the GO annotation terms that we used to select the genes shown in table 3 for characterization of the biological functions represented by the selected genes. Among the GO annotation terms associated with the genes selected in our meta-analysis, the GO-based gene set enrichment analysis highly ranked the cell cycle associated GO terms. The up-regulation of the expression of cell cycle genes in aggressive neuroblastoma tumours was already observed by Krasnoselsky et al. [26] in a comparison of tumours of different stages and *MYCN *amplification status, where patient outcome was not regarded. In addition to the cell cycle genes, gene set enrichment analysis of the GO terms associated with the significant genes resulted in high rankings of three other GO terms known to be affected in tumorigenesis: DNA damage response, negative regulation of MAPK activity [27] and Wnt receptor signalling pathway [28]. For DNA-damage response genes, higher expression can be observed in tumours with a unfavourable outcome than in tumours with favourable outcome. This effect can also be seen in other tumour entities like prostate cancer. For the gene *APEX1*, gene expression data is available in the gene expression data repository Oncomine [29] that shows increasing expression according to tumour malignancy (Additional File 1). This effect might be caused by accumulated genetic abberations in tumours with unfavourable outcome which trigger the activity of DNA-damage response genes.

While interpreting the analysis of gene expression for non-amplified advanced stage neuroblastoma tumours with regard to patient outcome, the influence of the therapy that all these patients have received has to be taken into account, as differences of the gene expression profiles with regard to patient outcome may not only reflect tumour malignancy but also tumour responsiveness to the therapy.

Although only a small number of patients were available for generation and assessment of a predictive model, outcome prediction based on data of both studies (only genes common to both platforms) yielded good results. Both patients with favourable and unfavourable outcome were classified with good results as indicated by a balanced ratio of sensitivity and specificity. This shows the potential of the approach to use data of different gene expression studies to derive predictive models for patient subgroups where gene expression data is rare. However, for stable and compact (in terms of the number of used genes) predictive models, a larger total number of samples is needed [30] which could be realised by combination of future gene expression profiling studies with the approach used here.

## Conclusion

In conclusion, cross-study analysis of gene expression data enables to detect consistent effects of gene expression based on completely independent data sets. The increase of statistical power through cross-study analysis is especially beneficial for the analysis of important patient subgroups for which gene expression data is rare. The presented results characterise a patient outcome specific set of differentially expressed genes in *MYCN *non-amplified advanced stage tumours. The resemblance of the expression of these genes in the investigated subgroup and in tumours with clearly defined (and diverging) outcome suggests that neuroblastomas comprise only two distinct biological subtypes associated with contrasting patient outcome.

Further investigations are needed to extend and validate these findings, a process in which the combination of future gene expression profiling studies which are larger both with respect to the investigated patients and probes on the utilised microarray platforms (thus yielding a large intersection of common probes), could play an important role.

## Competing interests

The author(s) declare that they have no competing interests.

## Authors' contributions

PW conceived of the study, carried out the analyses and drafted the manuscript. BB coordinated the study, participated in its design and helped to draft the manuscript. AO investigated the clinical records of the considered patients, contributed to the interpretation of the results and helped to draft the manuscript. MF and FW contributed to the interpretation of the results and helped to draft the manuscript. RE participated in the design of the study and helped to draft the manuscript. All authors read and approved the final manuscript.

## Pre-publication history

The pre-publication history for this paper can be accessed here:



## Supplementary Material

Additional File 1 Supplementary Information. Document giving further supplementary information. Click here for file

Additional File 2 Supplementary Figure 1. Hierarchical clustering of the expression data for the significant genes in advanced stage *MYCN *amplified tumors versus low stage non-amplified tumors from the study of Oberthuer et al. [8]. All samples with outcome information at 5 years after initial diagnosis have been used. The colored bar at the top of the figure denotes the status of outcome: black, fatal outcome; grey, favourable outcome. The colors refer to high (red) or low (green) expression relative to gene-wise means. Genes have been ordered by hierarchical clustering (correlation distance, complete linkage algorithm; dendrogram not shown). Click here for file

Additional File 3 Supplementary Figure 2. Hierarchical clustering of the expression data for the significant genes in advanced stage *MYCN *amplified tumors versus low stage non-amplified tumors from the study of Ohira et al. [7]. All samples with outcome information at 5 years after initial diagnosis have been used. The colored bar at the top of the figure denotes the status of outcome: black, fatal outcome; grey, favourable outcome. The colors refer to high (red) or low (green) expression relative to gene-wise means. Genes are shown in the same order as in Suppl. Fig. 1. Click here for file

Additional File 4 Supplementary Table 1. Genes associated with the GO categories mentioned in Tab. 3 (main manuscript). Click here for file

Additional File 5 Supplementary Figure 3. Venn diagram for comparison of the gene lists obtained by meta analysis („Meta analysis“), used for cross-platform classification („CPC“) or used for classification by Oberthuer et al. [8] („Oberthür“). Click here for file
