# Supplementary material for: Cross-study analysis of gene expression data for intermediate neuroblastoma identifies two biological subtypes
Source: BMC Cancer. 2007 May 25;7:89. doi: 10.1186/1471-2407-7-89 (PMC1904223; doi:10.1186/1471-2407-7-89)
Supplement: Additional File 1 — Supplementary Information. Document giving further supplementary information. [file 1471-2407-7-89-S1.pdf]

Cross-study analysis of gene expression data for intermediate neuroblastoma identifies two biological subtypes

Supplementary information

Risk stratification of neuroblastoma patients

Low-Risk Group				
**Country**	**Stage**	**Age**	**Mycn**	**other**
**Germany**	1,4S	any	normal	
	2	any	normal	1p normal
	3	< 2y	normal	1p normal
**Japan**	1,2,3,4S	< 1y	normal	
	1,2	> 1y	normal	

Intermediate-Risk Group				
**Country**	**Stage**	**Age**	**Mycn**	**other**
**Germany**	2,3	any	normal	1p deleted
	3	> 2y	normal	
	4	< 1y	normal	
**Japan**	3	> 1y	normal	
	4	< 1y	normal	

High-Risk Group				
**Country**	**Stage**	**Age**	**Mycn**	**other**
**Germany**	any	any	amplified	
	4	> 1y	any	
**Japan**	any	any	amplified	
	4	> 1y	any	

Supplemental Table 2 Comparison of risk stratification strategies in Germany and Japan. Risk stratification of neuroblastoma is different only for a small fraction of patients between Japan and Germany [3].

Identification of significant genes by meta-analysis.

To detect significant differential expression of a gene between the two outcome patient groups across studies, we applied a meta-analysis approach as described by Choi et al. [10]. For each gene in every study i the standardized mean difference between patient groups was calculated as an effect size $d_i = (\bar{X}_{ai} - \bar{X}_{di}) / S_{pi}$, where $\bar{X}_{ai}$ and $\bar{X}_{di}$ represent the means of the group of patients alive five years after diagnosis and the group of patients dead five years after diagnosis, respectively, and S_{pi} is the pooled standard deviation. A test statistic Q was used to decide whether a fixed effects model

(FEM) or a random effects model (REM) is more appropriate to combine the effect sizes of the different studies. A FEM assumes that the effect sizes (here, the standardized mean differences) observed in the different studies are samples of the same distribution. A REM explicitly accounts for differences between the studies by postulating that each effect size is drawn from a distribution with study specific parameters. Under the assumption that the differences in the effect sizes between studies is due to sampling error alone, the values for Q are distributed according to a χ^2 distribution. A quantile-quantile plot of the observed values of Q and the quantiles of a χ_1^2 distribution is shown in Supplemental Figure 1. As the observed Q values clearly deviate from the χ_1^2 distribution, the appropriateness of a random effects model is indicated, as the random effects model accounts for between-study variability.

Study-specific effect sizes were then combined in order to estimate the average effect size μ as described by Choi et al. [10]. Genes were chosen by comparing the effect size estimates with a given threshold and estimating the statistical significance with the concept of false discovery rate (FDR) based on empirical null distributions generated by random permutations (see Choi et al. [10] for details).

In order to visualise the expression of the significant genes, colour maps were generated using the R (http://www.r-project.org) function 'heatmap'. For hierarchical clustering we used an euclidean distance function and a complete linkage algorithm (as implemented in the R functions 'hdist' and 'hclust') on expression data transformed to zero mean and unit variance.

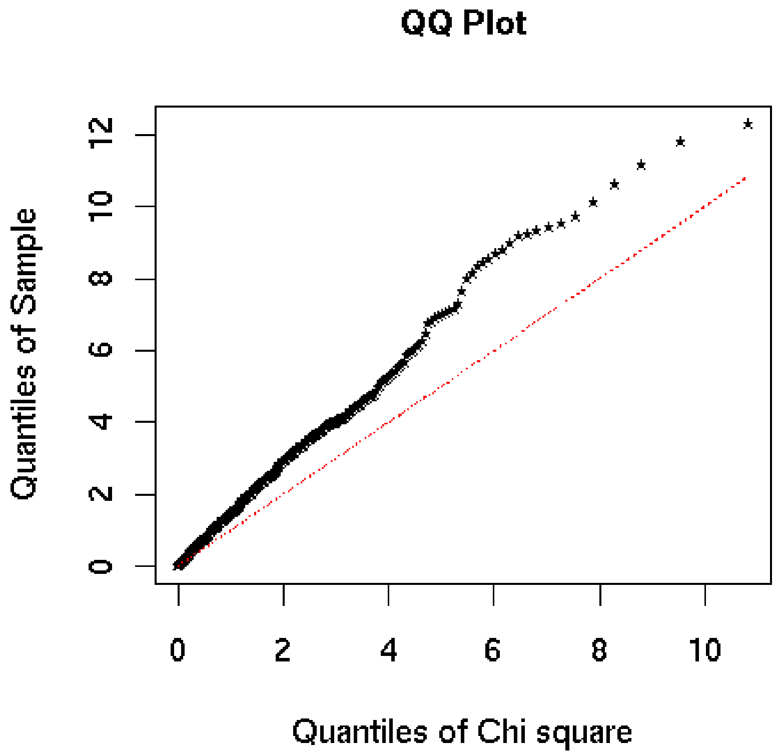

Supplemental Figure 1 QQ plot of the observed versus the expected quantiles of Q for the data of both neuroblastoma studies investigated. The expected Q values are from the χ_1^2 distribution.

Classification analysis.

A predictive model for the patient outcome status (alive vs. dead) five years after diagnosis was generated using the method of nearest shrunken centroids classification (also known as "Prediction Analysis of Microarrays", PAM) [18].

Using only the data of genes represented on both microarray platforms (n=1271), we applied a methodology evaluated by Warnat et al. [11], namely the median rank scores (MRS), to derive numerically comparable quantities from the expression values of both platforms used in the different studies. For application of the MRS method, the cDNA data of Ohira et al. was used as reference set. Then, nine patients of each prognosis group of both studies were selected as a training set to generate a predictive model of patient outcome. Thus, the training set consisted of 36 patients in total, 18 patients with favourable and 18 patients with unfavourable prognosis. The remaininig 30 patients were used as an independent test set. To generate a predictive model for patient outcome, the shrinkage parameter delta of the PAM method was optimized in a 3-fold cross-validation on the training set by selecting the value

yielding a minimal cross-validation error. Using the selected value for delta, a predictive model utilizing expression values of 256 genes was fitted on the training set and used to predict the outcome status of the samples in the independent test set. In addition, samples of the independent test set were classified according to the clinical markers age at diagnosis and INSS stage. The independent test set consisted of the following patients: S075, S089, S091, S093, S080, NB030, NB106, NB145, NB151, NB178, NB330, NB640, NB111, NB373, NB554, NB651, NB026, NB069, NB572, NB005, NB070, NB095, NB102, NB250, NB610, NB412, NB025, NB054, NB147, NB277.

Expression of the DNA-damage response gene *APEX1* in prostate cancer Supplemental Figure 2

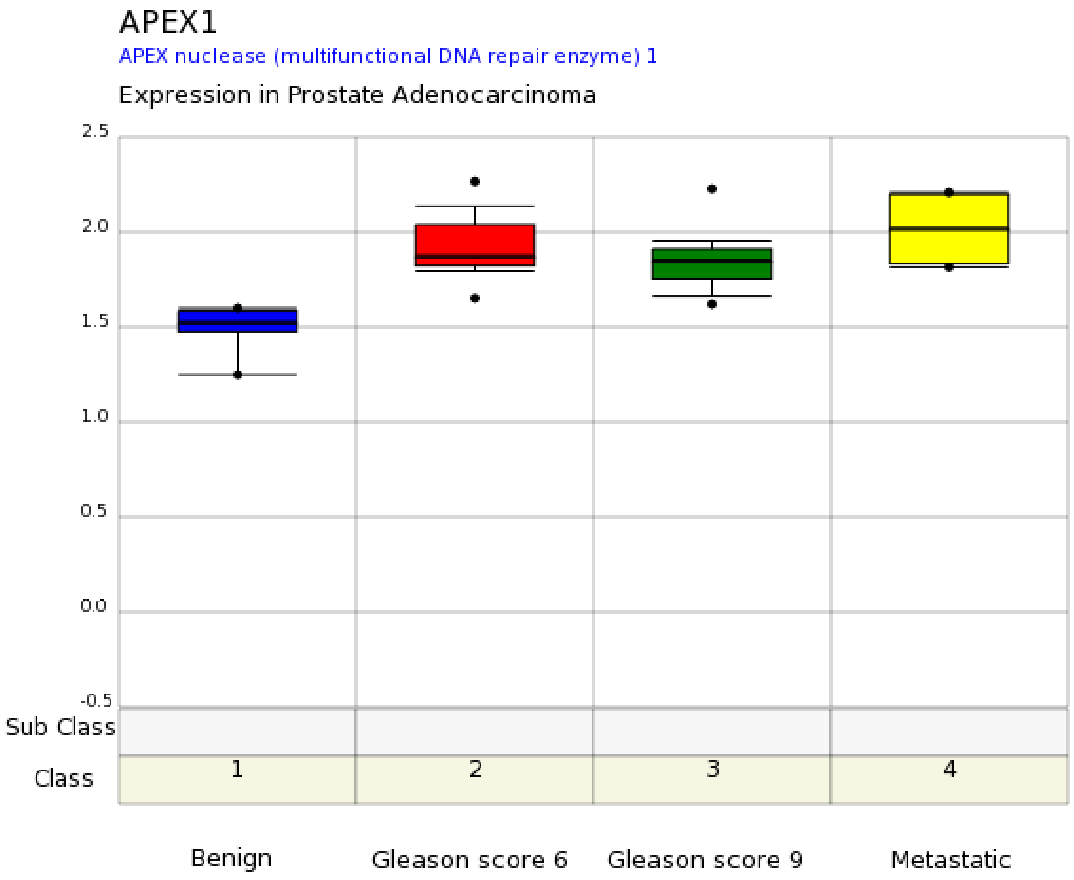

Expression of *APEX1* in prostate cancer [29]
