# Supplementary material for: Cross-study analysis of gene expression data for intermediate neuroblastoma identifies two biological subtypes
Source: BMC Cancer. 2007 May 25;7:89. doi: 10.1186/1471-2407-7-89 (PMC1904223; doi:10.1186/1471-2407-7-89)
Supplement: Additional File 2 — Supplementary Figure 1. Hierarchical clustering of the expression data for the significant genes in advanced stage MYCN amplified tumors versus low stage non-amplified tumors from the study of Oberthuer et al. [8]. All samples with outcome information at 5 years after initial diagnosis have been used. The colored bar at the top of the figure denotes the status of outcome: black, fatal outcome; grey, favourable outcome. The colors refer to high (red) or low (green) expression relative to gene-wise means. Genes have been ordered by hierarchical clustering (correlation distance, complete linkage algorithm; dendrogram not shown). [file 1471-2407-7-89-S2.pdf]

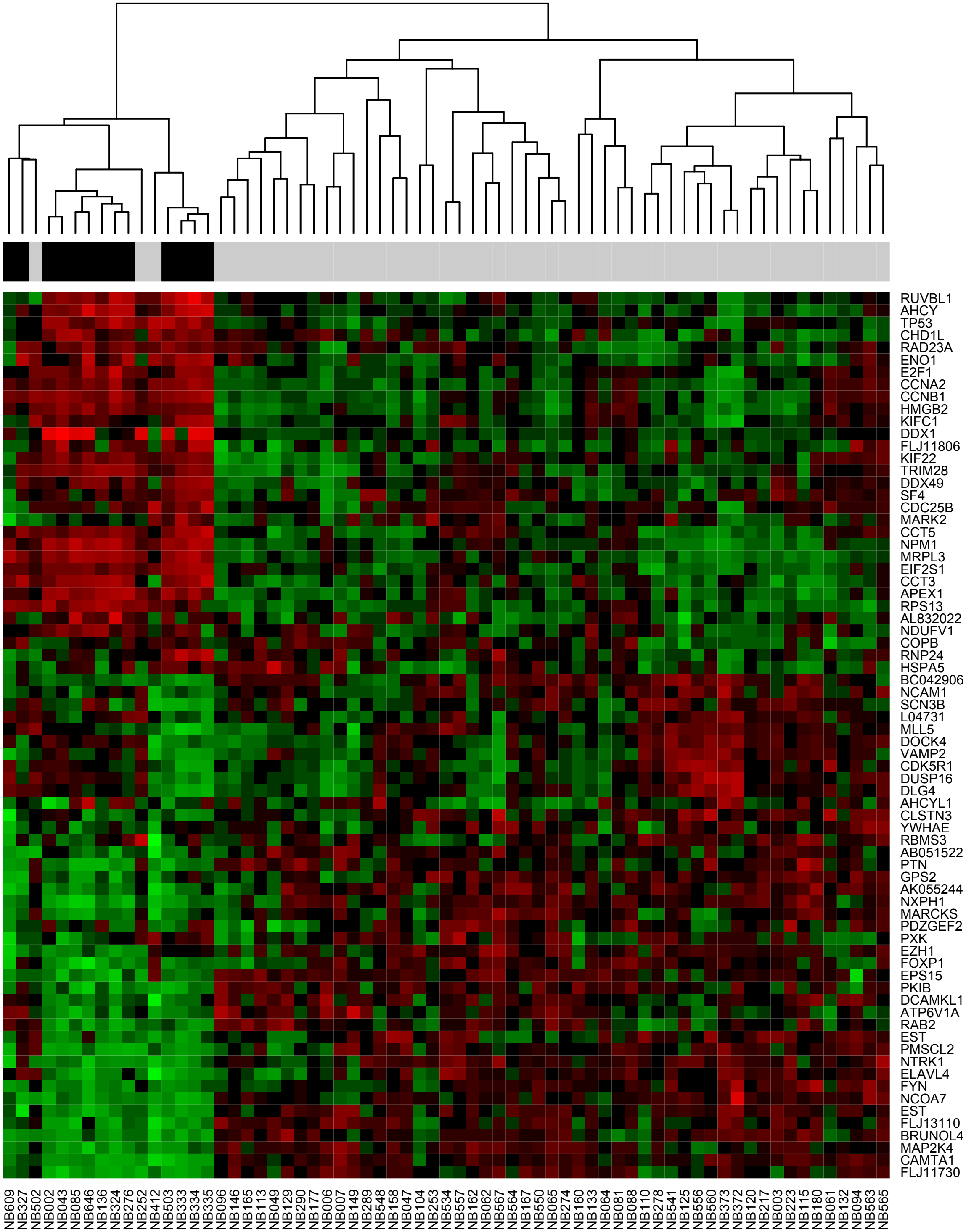
RUVBL1
AHCY
TP53
CHD1L
RAD23A
ENO1
E2F1
CCNA2
CCNB1
HMGB2
KIFC1
DDX1
FLJ11806
KIF22
TRIM28
DDX49
SF4
CDC25B
MARK2
CCT5
NPM1
MRPL3
EIF2S1
CCT3
APEX1
RPS13
AL832022
NDUFV1
COPB
RNP24
HSPA5
BC042906
NCAM1
SCN3B
L04731
MLL5
DOCK4
VAMP2
CDK5R1
DUSP16
DLG4
AHCYL1
CLSTN3
YWHAE
RBMS3
AB051522
PTN
GPS2
AK055244
NXPH1
MARCKS
PDZGEF2
PXK
EZH1
FOXP1
EPS15
PKIB
DCAMKL1
ATP6V1A
RAB2
EST
PMSCL2
NTRK1
ELAVL4
FYN
NCOA7
EST
FLJ13110
BRUNOL4
MAP2K4
CAMTA1
FLJ11730
NB609
NB327
NB502
NB002
NB043
NB085
NB646
NB136
NB324
NB276
NB252
NB412
NB503
NB333
NB334
NB335
NB096
NB146
NB165
NB113
NB049
NB129
NB290
NB177
NB006
NB007
NB149
NB289
NB548
NB158
NB047
NB104
NB253
NB534
NB557
NB162
NB062
NB567
NB564
NB167
NB550
NB065
NB274
NB160
NB133
NB064
NB081
NB088
NB110
NB278
NB541
NB125
NB556
NB560
NB373
NB372
NB120
NB217
NB003
NB223
NB115
NB180
NB061
NB132
NB094
NB563
NB565
