# Supplementary material for: Cross-study analysis of gene expression data for intermediate neuroblastoma identifies two biological subtypes
Source: BMC Cancer. 2007 May 25;7:89. doi: 10.1186/1471-2407-7-89 (PMC1904223; doi:10.1186/1471-2407-7-89)
Supplement: Additional File 3 — Supplementary Figure 2. Hierarchical clustering of the expression data for the significant genes in advanced stage MYCN amplified tumors versus low stage non-amplified tumors from the study of Ohira et al. [7]. All samples with outcome information at 5 years after initial diagnosis have been used. The colored bar at the top of the figure denotes the status of outcome: black, fatal outcome; grey, favourable outcome. The colors refer to high (red) or low (green) expression relative to gene-wise means. Genes are shown in the same order as in Suppl. Fig. 1. [file 1471-2407-7-89-S3.pdf]

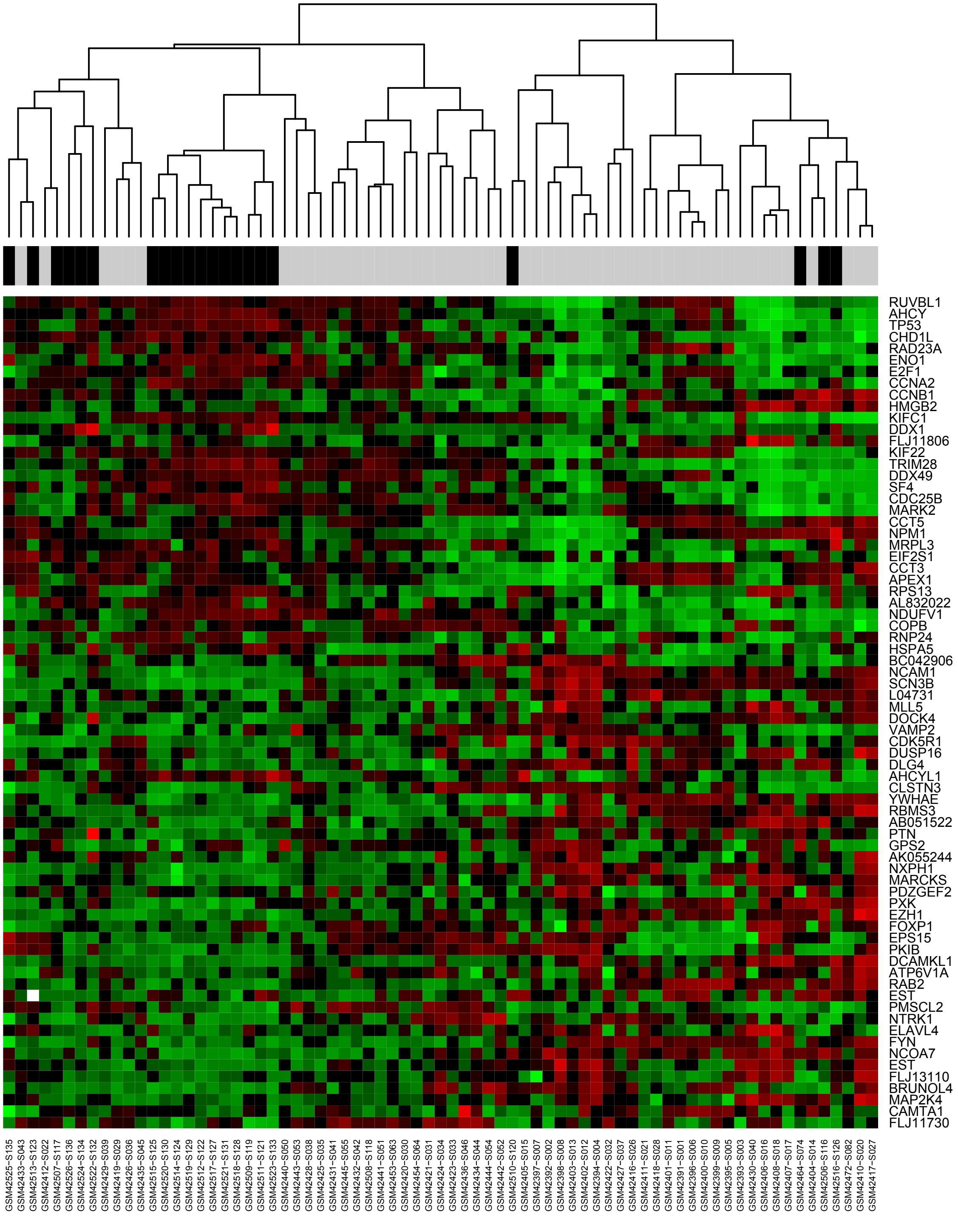
RUVBL1
AHCY
TP53
CHD1L
RAD23A
ENO1
E2F1
CCNA2
CCNB1
HMGB2
KIFC1
DDX1
FLJ11806
KIF22
TRIM28
DDX49
SF4
CDC25B
MARK2
CCT5
NPM1
MRPL3
EIF2S1
CCT3
APEX1
RPS13
AL832022
NDUFV1
COPB
RNP24
HSPA5
BC042906
NCAM1
SCN3B
L04731
MLL5
DOCK4
VAMP2
CDK5R1
DUSP16
DLG4
AHCYL1
CLSTN3
YWHAE
RBMS3
AB051522
PTN
GPS2
AK055244
NXPH1
MARCKS
PDZGEF2
PXK
EZH1
FOXP1
EPS15
PKIB
DCAMKL1
ATP6V1A
RAB2
EST
PMSCL2
NTRK1
ELAVL4
FYN
NCOA7
EST
FLJ13110
BRUNOL4
MAP2K4
CAMTA1
FLJ11730
GSM42525-S135
GSM42433-S043
GSM42513-S123
GSM42412-S022
GSM42507-S117
GSM42526-S136
GSM42524-S134
GSM42522-S132
GSM42429-S039
GSM42419-S029
GSM42426-S036
GSM42435-S045
GSM42515-S125
GSM42520-S130
GSM42514-S124
GSM42519-S129
GSM42512-S122
GSM42517-S127
GSM42521-S131
GSM42518-S128
GSM42509-S119
GSM42511-S121
GSM42523-S133
GSM42440-S050
GSM42443-S053
GSM42428-S038
GSM42425-S035
GSM42431-S041
GSM42445-S055
GSM42432-S042
GSM42508-S118
GSM42441-S051
GSM42453-S063
GSM42420-S030
GSM42454-S064
GSM42421-S031
GSM42424-S034
GSM42423-S033
GSM42436-S046
GSM42434-S044
GSM42444-S054
GSM42442-S052
GSM42510-S120
GSM42405-S015
GSM42397-S007
GSM42392-S002
GSM42398-S008
GSM42403-S013
GSM42402-S012
GSM42394-S004
GSM42422-S032
GSM42427-S037
GSM42416-S026
GSM42411-S021
GSM42418-S028
GSM42401-S011
GSM42391-S001
GSM42396-S006
GSM42400-S010
GSM42399-S009
GSM42395-S005
GSM42393-S003
GSM42430-S040
GSM42406-S016
GSM42408-S018
GSM42407-S017
GSM42464-S074
GSM42404-S014
GSM42506-S116
GSM42516-S126
GSM42472-S082
GSM42410-S020
GSM42417-S027
