# Supplementary figures and images for: Cross-study analysis of gene expression data for intermediate neuroblastoma identifies two biological subtypes
Source: BMC Cancer. 2007 May 25;7:89. doi: 10.1186/1471-2407-7-89 (PMC1904223; doi:10.1186/1471-2407-7-89)

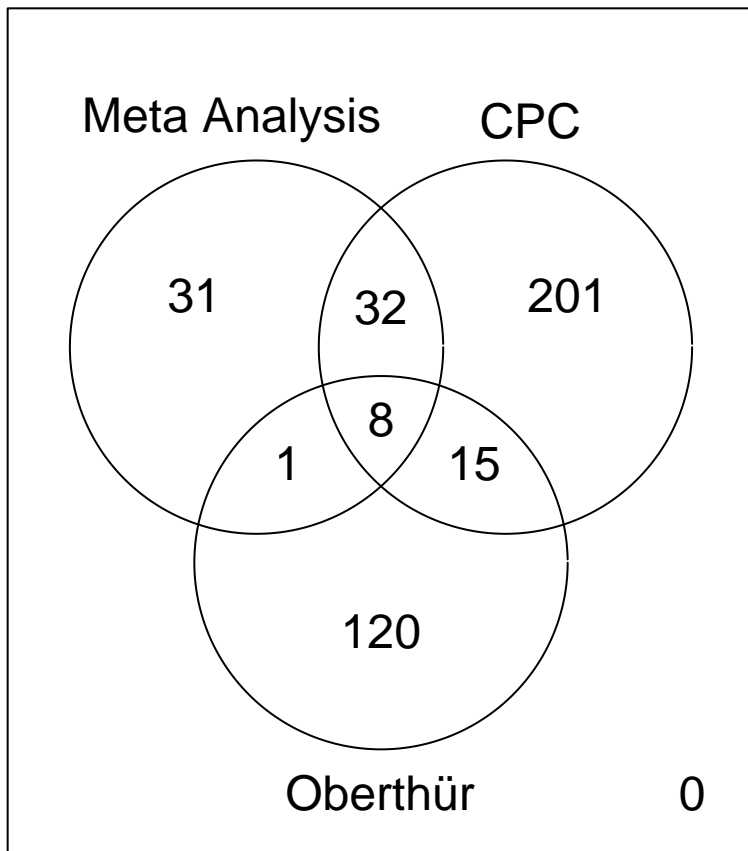

Supplement: Additional File 5 — Supplementary Figure 3. Venn diagram for comparison of the gene lists obtained by meta analysis („Meta analysis“), used for cross-platform classification („CPC“) or used for classification by Oberthuer et al. [8] („Oberthür“). [file 1471-2407-7-89-S5.pdf]
